# Antidepressants for chronic pain management: considerations from predictive modeling and personalized medicine perspectives

**DOI:** 10.3389/fpain.2024.1359024

**Published:** 2024-02-07

**Authors:** Xinyue Liu, Maja R. Radojčić, Ziye Huang, Baoyi Shi, Ge Li, Lingxiao Chen

**Affiliations:** ^1^ Department of Chinese Materia Medica, Tianjin University of Traditional Chinese Medicine, Tianjin, China; ^2^ Division of Psychology and Mental Health, Faculty of Biology, Medicine and Health, University of Manchester, Manchester, United Kingdom; ^3^ Department of Biostatistics, Mailman School of Public Health, Columbia University, New York, NY, United States; ^4^ Department of Public Health, Tianjin University of Traditional Chinese Medicine, Tianjin, China; ^5^ Department of Orthopaedics, Shandong University Centre for Orthopaedics, Qilu Hospital, Cheeloo College of Medicine, Shandong University, Jinan, Shandong, China; ^6^ Department of Biostatistics, School of Public Health, Cheeloo College of Medicine, Shandong University, Jinan, Shandong, China; ^7^ Sydney Musculoskeletal Health, School of Health Sciences, Faculty of Medicine and Health, University of Sydney, Sydney, NSW, Australia

**Keywords:** chronic pain, antidepressants, clinical prediction model, outcome measurement time, comorbid depression

## 1 Introduction

Chronic pain is one of the leading causes of disability (1, 2). Although non-pharmacological treatments are prioritized, the management of chronic pain commonly involves the utilization of pharmacological treatments. The 2021 National Institute for Health and Care Excellence (NICE) guideline for the management of chronic primary pain recommends antidepressants as their off-label use (3). The recommended medications are tricyclic amitriptyline, selective serotonin reuptake inhibitors citalopram, fluoxetine, paroxetine and sertraline, and serotonin-norepinephrine reuptake inhibitor duloxetine. The committee stated that the evidence of antidepressant superiority over placebo on improved quality of life, pain, sleep, and psychological distress was limited, mostly concerning the quality and amount of evidence, and to women with fibromyalgia. The 2020 NICE guideline for the management of neuropathic pain also recommended the use of antidepressants (4). However, previous studies showed that 56% of patients with chronic pain may not achieve adequate treatment effects when using antidepressants (5, 6). Hence, it is essential to identify these patients in advance, to facilitate resource allocation and reduce potential harm from inappropriate use. Clinical predictive modeling studies have been widely used to estimate treatment outcomes and optimize treatment strategies (7–9). Although several clinical predictive modeling studies have focused on treatment outcomes of pharmacological interventions for chronic pain, these studies are about nonsteroidal anti-inflammatory drugs rather than antidepressants (10, 11). To improve chronic pain management, in this article, we provide some insights for future clinical predictive modeling studies focusing on treatment outcomes of antidepressants for chronic pain, such as the possible impacts of comorbid depression and the choice of outcome measurement time on model development and evaluation of model performance.

## 2 Comorbid depression as a predictor

Patients with chronic pain more often have comorbid depression than those without pain (12, 13). However, the risk varies in different subgroups. For instance, patients who are female or obese are more likely to have comorbid depression. In addition, a previous study found that among people with chronic pain, pain relief following antidepressant use is over 30% related to the improvement of comorbid depression by antidepressants (14). Firstly, this indicates a potential for personalized management of chronic pain. Secondly, it points out the need to improve methods of addressing depression in future studies to provide stronger and more precise evidence for personalization. Therefore, we recommend mutual assessment of pain and depression at all study time points, considering comorbid pain and depression as a predictor category, and investigating the time-dependent relationship between pain and depression such as dual trajectory modeling. Furthermore, the Initiative on Methods Measurement and Pain Assessment in Clinical Trials (IMMPACT) emphasizes that comprehensive consideration of patient phenotypes is critical in predicting analgesic treatment outcomes (15). The IMMPACT mentioned that psychosocial factors (e.g., depression) are important phenotypic characteristics in predicting analgesic treatment outcomes and recommended measuring the overall severity of the depression (15). However, a recent study by Ebrahimi et al. has shown that there are significant individual differences in specific symptoms (e.g., irritability and anhedonia) among depressed patients with the same diagnosis and the same overall severity (16). It is indicated that focusing only on the overall severity of depression may not be sufficient and that the specific symptoms of depression (e.g., irritability and anhedonia) also need to be considered. Similarly, there are distinct relationships between certain antidepressants and specific symptoms of depression. For example, citalopram showed the highest efficacy in treating core mood symptoms, followed by sleep problems, and atypical symptoms (17), and escitalopram showed superior efficacy compared to nortriptyline in addressing emotional and cognitive symptoms (18). These findings indicated that comprehensively assessing depression, i.e., a personalized approach, can lead to selecting the most appropriate antidepressant for the given patients. Future research on antidepressants should include a more specific assessment of depression and pain specialists should be further educated on this matter.

## 3 The choice of outcome measurement time

Inappropriate choice of outcome measurement time may lead to invalid or inaccurate outcomes, which could affect model performance (19–21). In the following part, we mainly discuss (1) the establishment of the exposure time window and (2) the changing trend of the outcome.

### 3.1 The establishment of the exposure time window

#### 3.1.1 The definition

The exposure time window refers to the time from the completion of the causal effect of the exposure on the outcome that can be identified/observed (21). Using chest pain as an example, i.e., isosorbide dinitrate to relieve angina, the exposure time window refers to the time from when isosorbide dinitrate has improved the imbalance between the supply and demand of oxygen (when the causal effect of the exposure on the outcome has been established) until the patient experiences a reduction in pain symptoms (when the outcome can be observed) (22). This period should be selected based on the exposure and outcome of interest and the biological processes between the exposure and the outcome, i.e., by considering pharmacokinetics, pharmacodynamics, and pathophysiological pathways (23).

#### 3.1.2 The outcome measurement time falls within the exposure time window

Two possible biases should be noted (21). The first is that the measured outcome is invalid because the exposure-related outcome cannot yet be identified/observed, which occurs mainly in studies focusing on efficacy. For example, the efficacy of isosorbide dinitrate in alleviating angina symptoms is often observed over a time frame of around 30–60 min (24). Measuring angina relief within the first 30 min is prone to introducing invalid bias, as the true outcome cannot be adequately observed during this early period. The second bias is a misjudgment of the association between the exposure and the outcome, which occurs when continuous exposure terminates due to the appearance of early symptoms of the outcome before the diagnosis is identified. For example, the use of oral contraceptives, early symptoms like breast pain and tenderness, and the diagnosis of benign breast disease (BBD). When patients experience early symptoms, many are advised by their physicians to discontinue the use of contraceptives for breast-related reasons (25). Some case-control studies have shown that contraceptive use was less associated with BBD, leading to the conclusion that oral contraceptives may help prevent benign breast disease (26, 27). However, the protective effect of oral contraceptives on the development of BBD may be misjudged due to the second bias (28). This type of bias occurs mainly in the causal inference area and is not the subject of this paper. However, the first bias can occur when antidepressants are used to treat chronic pain. Although there is no consensus on the optimal time for measuring outcomes following the use of antidepressants for chronic pain, the recent NICE guidelines mentioned that outcomes can be measured after a period of 4–6 weeks of receiving antidepressants (3). Using the guideline as a reference, in a meta-analysis of the efficacy and safety of antidepressants for back pain and osteoarthritis, we found that five out of 33 (15%) included trials measured outcomes at or before week 4, which may lead to invalid results (29). This implies that the choice of outcome measurement time is influenced by the onset time of the medication, which depends on pharmacological, physiological, and pathological factors (30). The influence of pharmacological factors can be manifested in the mechanism and administration route. For example, esketamine nasal spray can take effect within four hours (31), far faster than the onset time of a typical antidepressant oral tablet (32). This may be related to the more direct mechanism of esketamine (increases the release of brain-derived neurotrophic factor) and the higher bioavailability when administered nasally. Therefore, when exploring the exposure time window, individual medications with their different mechanisms and administration routes should be considered to guide the optimal outcome measurement time.

The beginning point of the exposure time window is influenced by the exposure threshold (21), which is a certain level of exposure that needs to be reached before the benefits or risks associated with it begin to occur (23). Reaching the exposure threshold is related to the certain medication, its therapeutic dose and dosage regimen, i.e., the frequency of medication administration needed to achieve/maintain the therapeutic dose (21). The time to reach the threshold can vary depending on whether single or multiple doses are required (21, 24). If the threshold can be met with a single exposure, for example, using sublingual nitroglycerin tablets to relieve angina, one tablet at a time will normally relieve pain, meaning that a single dose is a complete exposure. If the threshold is met with multiple exposures, the exposure is not considered complete until all required doses have been given. There is still a lack of data on antidepressant exposure thresholds for treating chronic pain, which should be investigated further. In addition to medication factors, another point for consideration is pharmacogenetics, which influences the individual time needed to reach the therapeutic dose. Therefore, exposure thresholds are medication- and person-specific and lead to the difference in the time required for exposure completion, and consequently influence the beginning point of the exposure time window.

### 3.2 The changing trend of the outcome

The changing trend of the outcome can be discussed in three situations. The first is the changing nature of the outcome without/before the treatment; The second is the changing trend of the outcome due to the treatment effect; The third is the changing trend of the outcome after discontinuation of the treatment. Pain intensity changes during a day, a week, a month. Patients with musculoskeletal pain usually experience morning stiffness and pain, and to them, morning corresponds to the most intense pain, while neuropathic pain is the most troublesome during the night. On the other hand, individuals’ evening chronotypes have been associated with multi-site pain (33). A study that investigated daily musculoskeletal pain trajectories over a month found that approximately 40% of patients had unstable pain that varied more than 1 point from the monthly mean for three consecutive days (34). The same study also reported that 46% of participants were excluded as they did not comply with the protocol, mainly not reporting pain every day (34). Therefore, requesting more granular data results in decreased patient compliance which negatively impacts the quality of data collected and knowledge acquired. Granularity should not be a requirement for all research questions. While daily pain variations have great potential for personalized management, they are not helpful for drug effectiveness studies and prediction modeling. The personalization component assumes that the medication dosage regimen should be tailored to ensure the peak concentration is achieved when the patient's pain is the most intense. Following this, treatment options are expected to impact the outcome, as indicated in the second situation of the changing trends. The treatment effect can (1) provide initial pain relief, which would correspond to a trajectory of a short sharp decrease followed by an increase at a slower rate; (2) work better with each dose in relieving pain, corresponding to a trajectory of steady pain decrease; and (3) take time to achieve the benefit, in which case, the pain trajectory would initially be unchanged and after some time start slowly to decrease (23). Therefore, to observe these outcome trajectories and their possible variations, the outcome (pain) should be measured frequently and long enough.

Although we did not find relevant studies of antidepressants for chronic pain, there are several studies of analgesics for chronic pain. For example, Radojčić et al. investigated pain trajectories and responses to analgesic treatments (i.e., analgesics, nonsteroidal anti-inflammatory drugs, and steroids) among patients with knee osteoarthritis (OA) (35). This study identified four types of phenotypes: “low-fluctuating”, “mild-increasing”, “moderate-treatment-sensitive”, and “high-treatment-insensitive”. Importantly, they found a small proportion of knee OA patients who used analgesics, which did not improve their severe pain. Among other phenotypes, the effect of analgesics was observed to different degrees (35). This study used two data sources. The first data source measured outcomes at six-month intervals over three years, and the second data source measured outcomes at one-year intervals over nine years. Johnson et al. investigated pain trajectories in knee OA patients over 18 months, with pain outcomes measured every three months (36). Taken together, both studies demonstrated that patients with knee pain should be followed for more than three years to observe outcome changes. Finally, treatment is expected to reinstate the balance disturbed by the disease and provide a symptom-free period after its discontinuation. However, patients are rarely followed up after the discontinuation of the treatment, and post-treatment changes in the outcome, how long it takes patients to seek healthcare again and start new treatments are mostly unknown. These should be considered as indirect outcomes of treatment effectiveness and explored via prediction modeling.

## 4 Conclusion

To optimize the use of antidepressants for chronic pain and provide better evidence for future guidelines, we discussed two important but easily overlooked issues- comorbid depression and the appropriate time for outcome measurements. We indicated the importance of balancing between needed data granularity and patients’ compliance, given the research question and implications of the findings. Personalized medicine is the future of pain management, and to achieve the desired progress, we need to improve prediction modeling and optimally consider the specific nature of each medication and the measured outcome.
